# Examining the independent and combined effects of autistic and ADHD traits on multisensory integration

**DOI:** 10.3389/fnhum.2026.1824956

**Published:** 2026-06-15

**Authors:** Carolynn Hare, Michelle Luszawski, Samantha E. Schulz, Yassmine Arnaout, Ryan A. Stevenson

**Affiliations:** 1 Department of Psychology, Western University, London, ON, Canada; 2 Centre for Brain and Mind, Western University, London, ON, Canada; 3 Western Institute for Neuroscience, Western University, London, ON, Canada; 4 Department of Psychology, Carleton University, Ottawa, ON, Canada; 5 Neuroscience Program, Western University, London, ON, Canada

**Keywords:** attention-deficit/hyperactivity disorder (ADHD), audiovisual, autism spectrum disorder, multisensory integration (MSI), research domain criteria, sensory detection threshold

## Abstract

Multisensory integration differences have been reported in individuals with Autism Spectrum Disorder (ASD) and Attention-Deficit/Hyperactivity Disorder (ADHD), yet little is known about how these traits may jointly influence multisensory processing. Given the high comorbidity between ASD and ADHD, examining their individual and combined effects is critical for understanding sensory integration in neurodivergent populations. The present study examined associations between Autistic and ADHD traits and multisensory integration in a broad university sample of 92 young adults (62 females, *M_age_* = 18.29, age range = 17–24). Participants completed a speeded detection task to assess multisensory gain and self-report measures of Autistic and ADHD traits. Autistic and ADHD traits were not associated with accuracy gain. No evidence was found for an interactive or additive effect of co-occurring Autistic and ADHD traits on multisensory integration. These findings suggest that multisensory integration in young adults may be largely independent of Autistic and ADHD trait expression when perceptual sensitivity is controlled. This highlights the importance of accounting for individual sensory thresholds when studying multisensory processing in neurodivergent populations, and suggests that differences in multisensory integration may be more strongly influenced by diagnostic status or developmental stage than by trait variation alone.

## 1 Introduction

Autism Spectrum Disorder (ASD) and Attention-Deficit Hyperactivity Disorder (ADHD) are highly comorbid neurodevelopmental conditions. Between 50 to 70% of Autistic individuals present with comorbid ADHD (Hours et al., 2022) and 21% of ADHD individuals present with comorbid ASD (Hollingdale et al., 2020). ASD is a neurodevelopmental condition characterized by deficits in social communication and restricted, repetitive sensory–motor behaviours (American Psychiatric Association, 2013). ADHD is a neurodevelopmental condition that is characterized by the presence of inattentive and hyperactive–impulsive symptoms, with three presentations: inattentive (IA), hyperactive–impulsive (HI), and combined (C) (American Psychiatric Association, 2013). Although diagnostically different, ASD and ADHD share several overlapping features, including differences in executive functioning (Lukito et al., 2017), sensory processing challenges (American Psychiatric Association, 2013; Scheerer et al., 2024), and potentially shared genetic pathways (Elia et al., 2010; Williams et al., 2012). Further, neuroimaging evidence suggests that sensory characteristics and their underlying neurobiology are transdiagnostic in nature instead of specific to ASD or ADHD (Choi et al., 2025). Evidence suggests ASD and ADHD are related but distinct diagnoses, and their comorbid state may represent an “additive” profile for the two conditions (Antshel and Russo, 2019).

One of the most prominent areas of overlap between ASD and ADHD is atypical sensory processing. Individuals across both conditions frequently report hyper- or hypo-sensitivity to sensory input across modalities, including auditory and visual domains, as measured through questionnaire based, behavioural, and neural approaches (ASD: Ashwin et al., 2009; Bennetto et al., 2007; Jones et al., 2009; Larsson et al., 2017; Lepistö et al., 2008; Mikkelsen et al., 2018; Puts et al., 2014; Schauder and Bennetto, 2016; Schulz and Stevenson, 2019; ADHD: Bartgis et al., 2009; Dellapiazza et al., 2019; Dunn and Bennett, 2002; Ghanizadeh, 2011; Little et al., 2018; Lucker et al., 1996; Mangeot et al., 2007; Panagiotidi et al., 2018; Söderlund and Jobs, 2016). These sensory differences are clinically meaningful, given that sensory processing is a foundational building block upon which most higher-level cognitive processes rely.

Beyond unisensory sensitivity, sensory processing also includes the ability to merge information across sensory modalities, a process known as *multisensory integration* (Stein and Wallace, 1996). Multisensory integration allows individuals to process sensory information in a more efficient manner, resulting in improved behavioural and perceptual performance, an effect often referred to as *multisensory gain*. Importantly, sensory sensitivity and multisensory integration are conceptually and mechanistically distinct processes: enhanced or reduced sensitivity to unisensory stimuli does not necessarily imply differences in how information is integrated across modalities.

Altered multisensory processing has been found in both ASD (Ainsworth et al., 2021; Bebko et al., 2014; Brandwein et al., 2013; Collignon et al., 2013; Foxe et al., 2015; Ostrolenk et al., 2019; Russo et al., 2010; Segers et al., 2020; Stevenson et al., 2014c, 2014d; Stevenson et al., 2017; Woynaroski et al., 2013; see Feldman et al., 2018 for review) and ADHD (Bisch et al., 2016; Hare et al., 2024; McCracken et al., 2019; Panagiotidi et al., 2017; Schulze et al., 2021, 2022). However, little research has examined the combined influence of ASD and ADHD on multisensory integration.

Multisensory integration can be measured by a wide range of paradigms, for example, speeded detection tasks and perceptual illusions, and it can be measured by using a range of stimuli, ranging from simpler (e.g., flashes, beeps) to more complex stimuli (e.g., speech). Findings across ASD and ADHD populations vary considerably depending on the type of stimuli and task demands. Given this heterogeneity, it is essential to delimit the present focus: the current work examines audiovisual integration in a speeded response-time detection paradigm using perception-matched simple stimuli (e.g., Gabor patches and beeps).

Autistic individuals show reduced multisensory integration compared to neurotypical individuals (Feldman et al., 2018; Noel et al., 2017; Ostrolenk et al., 2019; Stevenson et al., 2014d). Group differences are often more pronounced when higher-level or socially relevant stimuli, such as audiovisual speech, are used (Baum et al., 2015; Bebko et al., 2014; Beker et al., 2018; Foxe et al., 2015; Stevenson et al., 2014d; Stevenson et al., 2017), whereas findings using simple audiovisual stimuli are more mixed. Neurophysiological studies consistently demonstrate reduced neural indices of audiovisual integration in Autistic children and adults (Brandwein et al., 2011, 2013), but behavioural results from speeded detection tasks show variability across studies (Dwyer et al., 2022; Molholm et al., 2020; Ostrolenk et al., 2019).

One potential reason for the inconsistent results across behavioural paradigms has been how unisensory perception is handled. Studies that statistically control for unisensory perception thresholds often fail to find clear behavioural differences in audiovisual integration (Dwyer et al., 2022), whereas studies that perceptually equate auditory and visual detectability across participants, such as using adaptive staircase procedures, are more likely to observe reduced multisensory integration in ASD (Molholm et al., 2020). This distinction highlights a critical methodological issue: differences in sensory sensitivity may obscure or masquerade as differences in multisensory integration if perceptual thresholds are not controlled.

Autistic traits, reflecting the continuous distribution of characteristics that define clinical autism, are present throughout the general population traits (Constantino and Todd, 2003; Robinson et al., 2011; Ruzich et al., 2015). Despite growing interest in dimensional approaches, relatively little work has examined how autistic traits relate to audiovisual integration in speeded detection paradigms. One recent study reported slower responses to audiovisual stimuli in individuals with higher Autistic traits, but stimuli were not perception-matched (Poulain et al., 2023). Consequently, the findings were interpreted in terms of increased sensory reactivity rather than differences in multisensory integration (Poulain et al., 2023).

Compared to ASD, findings regarding multisensory integration in ADHD are more inconsistent. Studies have reported enhanced multisensory integration (Bisch et al., 2016; McCracken et al., 2019; Schulze et al., 2022), no difference (Hare et al., 2024; Schulze et al., 2021), or reduced multisensory integration (Hare et al., 2024; Michalek et al., 2014; Schulze et al., 2021) relative to neurotypical individuals, with larger effects often emerging for complex or speech-based stimuli (Hare et al., 2024; Schulze et al., 2021). In a study using a stimulus-matched speeded-response time task, ADHD adults showed a violation of the race model earlier in the response than neurotypical adults, which they interpreted as a marker for impulsivity (McCracken et al., 2019). There were also significant group differences in event-related potentials (ERPs) in frontal, parietal, and occipital brain regions, which are regions reported to be altered in those with ADHD. Building on these clinical findings, research has shifted to examining ADHD traits.

As with ASD, ADHD traits are continuously distributed in the population (Hudziak et al., 2007; Levy et al., 1997). This distribution supports the use of quantitative, dimensional approaches in studying general population samples (Chen et al., 2008; Levy et al., 1997; Paloyelis et al., 2010). To date, however, no known studies have examined the relationship between ADHD traits and multisensory integration using speeded response-time detection tasks in adults. Existing work in university samples has employed illusion-based or speech paradigms, with no observed associations between ADHD traits and multisensory integration in tasks such as the sound-induced flash illusion, the McGurk effect, and audiovisual speech-in-noise perception (Hare et al., 2026b). In contrast, more reliable associations have emerged for temporal aspects of multisensory processing, with higher ADHD traits linked to narrower temporal binding windows under specific task demands (Hare et al., 2026a; Panagiotidi et al., 2017). Hyperactive–impulsive traits compared to inattentive traits show some evidence of being more strongly related to multisensory integration and temporal processing differences (Hare et al., 2024). Together, this pattern suggests that temporal processing may be preferentially affected by higher ADHD traits, whereas other measures of multisensory integration (i.e., accuracy) may be less affected.

These findings suggest that multisensory integration is affected in both ASD and ADHD, albeit in different ways. Given the high comorbidity of ASD and ADHD, the combined effect of both diagnoses should be examined regarding multisensory integration. Overall, little research has investigated the combined effect of Autistic and ADHD traits on multisensory integration. To examine the similarities and differences of these conditions, along with their possible “additive profile,” we examined the individual and combined influence of Autistic and ADHD traits on multisensory integration in undergraduate students using the Research Domain Criteria Framework (RDoC; Cuthbert, 2014). RDoC supports the study of traits across the entire spectrum of a disorder, from levels found in the general population to clinically relevant levels. Further, it suggests examining different domains of functioning, such as sensory processing. Using this RDoC framework, we are in alignment with the push to investigate transdiagnostic, more dimensional considerations which may explain the etiological overlap and shared impairments and outcomes (Ameis, 2017; Kozak and Cuthbert, 2016). Overall, this work aims to use a dimensional approach to examine the unique and combined influence of Autistic and ADHD traits on the key sensory process of multisensory integration.

To accomplish this goal of examining how Autistic traits and ADHD traits, both individually and combined, are related to audiovisual multisensory integration we are using a well-validated perception-matched speeded detection task in a broad university population. The stimuli were visual Gabor patches and auditory beeps, both embedded in noise, and the multisensory stimuli was a combination of the auditory and visual stimuli that each participant can perceive 50% of the time determined via a staircasing procedure. This paradigm accounts for unisensory processing differences which are common in ADHD and Autistic individuals. The multisensory integration measure will be multisensory gain, the behavioural benefit associated with a multisensory stimulus compared to a unisensory stimuli, and was examined using response-time (i.e., Miller’s Race Model) and accuracy-based measures.

First, we examined whether overall and specific Autistic traits are related to multisensory gain. Specifically, we predicted that increased Autistic traits would be related to decreased multisensory gain, for both response-time and accuracy-based measures, in line with previous literature in non-clinical adults and Autistic adults (Kawakami et al., 2020; van Laarhoven et al., 2019).

Second, we examined whether overall and presentation ADHD traits are related to multisensory gain. Specifically, are inattention (IA), hyperactive–impulsive (HI), and combined (C) or total traits related to multisensory gain. We predicted that ADHD traits will be related to multisensory gain, specifically that larger multisensory gain would be related to higher ADHD traits, as previous studies show greater multisensory gain using response time-based measures (Hare, 2024; McCracken et al., 2019). Accuracy gain may follow a different pattern as some studies have shown no difference in accuracy gain or slight reductions in accuracy gain in ADHD individuals compared to NT individuals (Hare, 2024; Hare et al., 2024).

Third, we examined the additive effects of Autistic and ADHD traits on multisensory gain. We predicted an additive effect of Autistic and ADHD traits on multisensory gain, with a combination of high traits in both leading to reduced multisensory gain. This prediction reflects the possibility that although ADHD traits have been associated with enhanced multisensory integration in timing specific contexts, Autistic traits are more consistently associated with differences in multisensory integration and may place constraints on effective integration processes. As a result, the presence of elevated Autistic traits may attenuate or override ADHD-related increases in multisensory gain.

We expected that Autistic traits will have a stronger influence on multisensory integration as there are more consistent findings of multisensory integration differences in Autistic populations compared to ADHD populations. Additionally, given the high rate of comorbidity between autism and ADHD, previous studies of multisensory integration in Autistic samples may have included individuals with elevated ADHD traits unless these were explicitly screened or controlled. Another possibility is that opposing associations between Autistic and ADHD traits could contribute to reduced or null effects when both trait dimensions are considered simultaneously, potentially reflecting interactive rather than purely additive influences on multisensory gain.

## 2 Methods

### 2.1 Participants

One hundred and thirty participants were recruited from the university’s undergraduate research participation pool. Participants were excluded if they failed to reach both a visual and auditory threshold (between 40 and 60% accuracy) throughout the experiment or for not completing questionnaires, which resulted in a final sample of 92 participants (62 females, *M_age_* = 18.29, age range = 17–24). Participants had self-reported normal or corrected-to-normal hearing and vision, and all participants reported that they were fluent in English. Experimental protocols were approved by the university’s Non-Medical Research Ethics Board. This data partially overlaps with a previously reported study examining behavioural sensory sensitivity in the same participants (Schulz and Stevenson, 2022). This study used an RDoC framework approach; thus, all participants with and without an ADHD or ASD diagnosis were included in the study as they were eligible for inclusion to inform a broad range of the key variables (Cuthbert and Insel, 2013; Hengartner and Lehmann, 2017; National Institute of Mental Health, n.d.).

### 2.2 Stimuli

**Figure 1:**
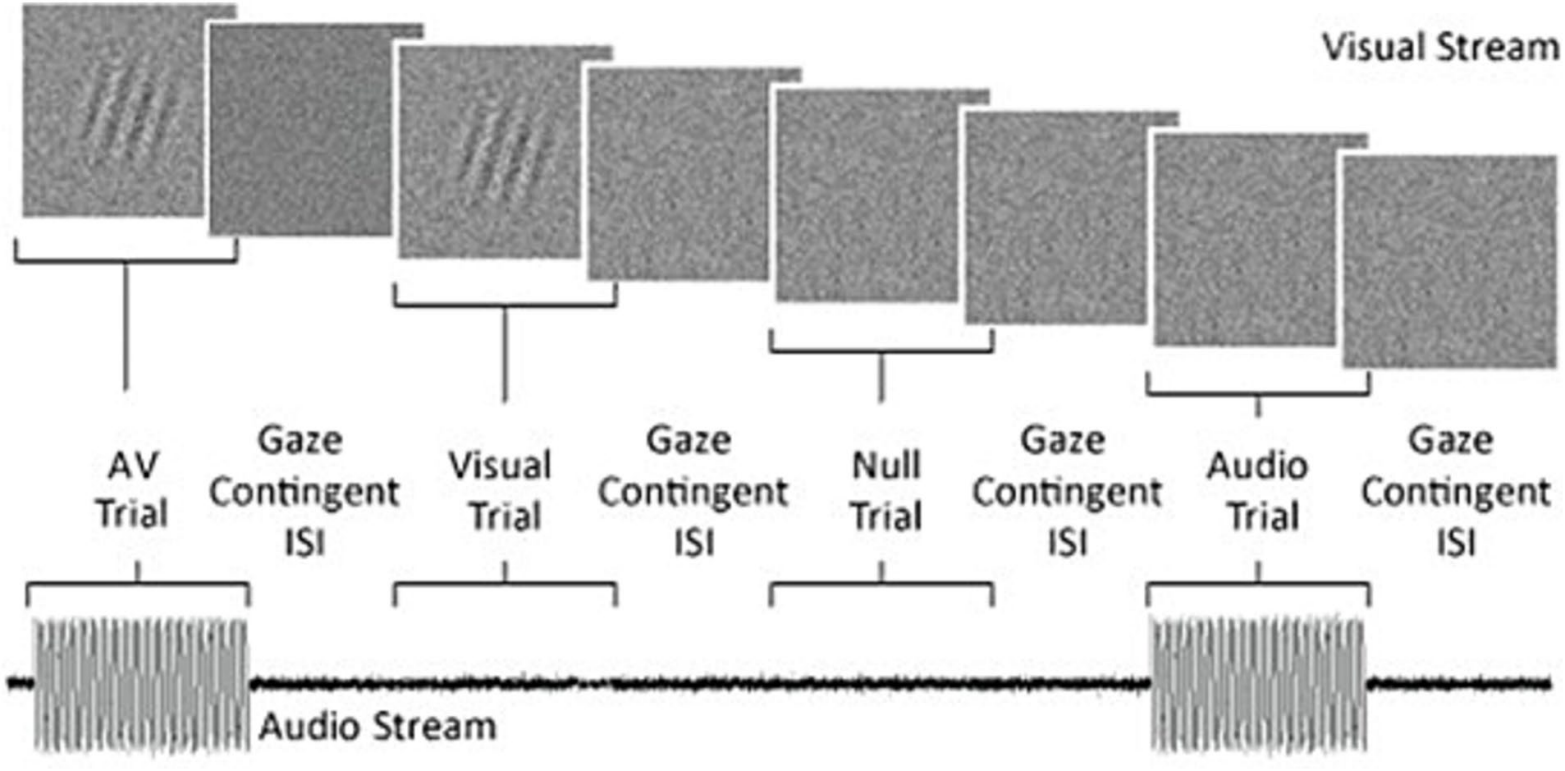
Trial procedure with example of unisensory and multisensory stimuli. The task was adapted from Schulz and Stevenson (2020), the figure is not adapted from that paper. Note that audiovisual trials were only presented during the second phase.

Each participant was presented with auditory and visual stimuli using E-Prime 3 (Psychology Software Tools, 2016) on the computer screen for 100 ms and refreshed at a rate of 16.67 ms (60 Hz). Gaze contingency was measured using Tobii Pro Extensions (Tobii, 2018). Auditory pure tones were presented at a frequency of 800 Hz within 40 decibel sound pressure level (dB SPL) of continual white noise. Tones ranged from 35 dB SPL to 69.5 dB SPL presented at every half decibel. Sound levels were verified using a sound pressure level meter. Visual stimuli included sinusoidal luminance gratings, known as Gabor patches, that were presented evenly based on Michelson contrast between 0.01 to 0.1, and positioned in static visual noise (see Figure 1 for example). Luminance was verified using a photometer. The Gabor patches subtended 9° of visual angle with a frequency of 30 cycles per degree. For each contrast, the patches were randomly oriented, excluding vertical and horizontal orientations.

### 2.3 Procedure

Participants were seated in a dark, sound-controlled room, 60 cm from a desktop computer (Lenovo ThinkCentre M710s, model: 0037US) monitor (Acer LCD Monitor, model: X223W). A chin rest was used to decrease head movement and verify all participants were at equal distance from the monitor and speakers. A Tobii Pro X3–120 eye-tracker was connected to the bottom of the computer monitor to gather gaze-contingent consistency across trials. A nine-point calibration of the eye-tracker was completed using the TET calibration package call (Tobii, 2018). Subsequently, the participant completed practice trials to become accustomed to the background light and noise and to familiarize themselves with the experimental stimuli.

The experiment consisted of two phases: (1) a detection task using an adaptive staircase and (2) and a speeded detection task. During the first phase, participants completed an adaptive psychophysical staircase procedure in which they were presented with unisensory auditory and visual stimuli to assess their sensitivity thresholds in each modality. In the second phase, visual, auditory, and audiovisual stimuli were presented within a small range around each participant’s thresholds, and the participants performed a speeded detection task (Schulz and Stevenson, 2020). Afterwords, participants filled out two questionnaires measuring ADHD and Autistic traits: ADHD Adult Self-Report Scale (ASRS) and Broad Autism Phenotype Questionnaire (BAPQ).

#### 2.3.1 Sensory detection staircase

In phase 1, the task consisted of 600 trials: 150 auditory, 150 visual, and 300 null trials. Auditory and visual white noise were presented throughout the entire experiment, with no clear trial boundaries apparent to the participants. Each trial began with a minimum of 900 ms of continued auditory and visual noise. Using gaze-contingent trial control, the task proceeded only after participants fixated on the screen for 200 ms. An auditory or visual stimulus was then presented in the continuous noise for 100 ms, or in the case of a null trial, auditory and visual noise continued for 100 ms, with no stimulus presentation. Following stimulus or null presentation, the noise remained on screen for up to 1,500 ms while responses were collected. During the task, participants were instructed to indicate the presence of any stimulus, either auditory or visual, by pressing the space bar as quickly and as accurately as possible. If a participant responded, the response window ended immediately and the next trial began seamlessly, with the continuous auditory and visual noise maintained throughout the task.

Sensory thresholds were calculated for each participant, quantified as the stimulus intensity that was accurately detected by the participant in 50 % of presentations. Of note, detection thresholds are one of two methods used for determining behavioural sensitivity thresholds. Alternatively, discrimination thresholds reveal a participant’s ability to detect minute changes in stimuli intensity. This task was used in previous research (Schulz and Stevenson, 2020, 2022).

Trials were presented in six interleaved one-up-one-down adaptive staircases; three in the auditory domain and three in the visual domain. If a participant accurately detected a stimulus upon presentation, the stimulus presented in the subsequent trial within the same staircase was reduced in intensity (increased difficulty of detection). Alternatively, if a participant failed to detect a stimulus upon presentation, the stimulus presented in the subsequent trial within the same staircase was increased in intensity (decreased difficulty of detection). Each staircase increased or decreased in difficulty by 8 levels until the first reversal. A reversal occurs when a missed presentation follows an accurately detected presentation, or an accurate detection follows a missed presentation. The reversal implies a shift from the stimuli intensity increasing in difficulty from the previous trial to decreasing in difficulty in the subsequent trial, or vice versa.

For example, in a staircase beginning with a low intensity stimulus (difficult to detect), the participant likely missed the first few stimuli presentations in a row as they are below the participant’s detection threshold, and with each missed trial, the intensity of the stimuli continued to increase (get easier) within the respective staircase. Once the staircase exceeded the participant’s detection threshold and the participant did not respond, the first reversal occurred, and the next stimulus was more intense (easier to detect). As the participant approached their threshold, reversals occurred on approximately 50 % of the trials, such that the participant is presented with a stimulus they are just barely able to detect, followed by an undetectable stimulus. After the first reversal, step size was reduced to 4 levels until the second reversal. After the fourth reversal, step size was reduced to 2 levels, until the sixth reversal, at which point, step size was reduced to 1 step per trial.

In the visual domain, staircases began at a Michelson contrast of 0.017 (difficult), 0.056 (moderately difficult), and 0.095 (easy). Likewise, in the auditory modality, staircases began at 37.5 (difficult), 52.5 (moderately difficult), and 67.5 (easy) dB SPL. Trials were presented within blocks of 12 trials. Each block consisted of one trial from each staircase, presented in a random order. As the experiment progressed, the three staircases in each domain converged and a threshold was established as the median level of the three staircases after six reversals were made within each staircase in each sensory domain. Thus, a threshold was determined independently in the auditory and visual domains and will be referred to as visual and auditory thresholds. Visual threshold was determined by the contrast at which they can detect the stimuli half of the time; therefore, lower thresholds are indicative of higher sensory sensitivity and vice versa. Likewise, an individual’s auditory threshold is the intensity (dB SPL) at which they can detect the stimulus half the time.

#### 2.3.2 Speeded detection task

In phase 2, the task was identical to phase 1 except for the addition of audiovisual trials. Audiovisual stimuli (*N* = 150) were randomly presented with auditory (*N* = 150), null (*N* = 450), and visual (*N* = 150) unisensory trials. For each participant, the auditory component of the audiovisual stimulus was presented at the individual’s previously determined auditory detection threshold (dB SPL). Similarly, the visual component in the audiovisual stimuli was displayed at the individual’s visual contrast (Michelson Contrast) detection threshold. Each participant was therefore presented with contrast and dB SPL values at their 50% detection threshold, thus ensuring the difficult level was equal for each participant. Thus, if a participant required low-intensity (i.e., difficult to detect) stimuli to reach threshold, the audiovisual condition was likewise presented at correspondingly low auditory and visual intensities. The participants were instructed to press the spacebar upon detection of the visual, auditory, or audiovisual stimuli as quickly and accurately as possible.

Multisensory integration studies generally use stimulus-matched paradigms in which participants are shown the same intensity stimuli. In this study, we controlled for individual differences in sensory sensitivity by presenting the stimulus at each individual’s perceptual level, which we will refer to as perception-matched. The perception-matched detection task will be matched to the sensory stimuli which each individual is able to perceive 50% of the time and will give us a measure of behavioural sensory sensitivity in both the visual and auditory domains. In this way, we will be able to account for individual differences in sensory sensitivities associated with Autistic or ADHD traits.

Specifically, a perception-matched design will be used because multisensory gain can be influenced by individual differences in unisensory sensitivity. When sensory signals are weak or hard to detect, larger multisensory benefits may emerge due to inverse effectiveness, whereas heightened sensitivity in one modality may also yield improved audiovisual performance through statistical facilitation rather than integrative processing (Meredith and Stein, 1986, 1996; Schneeberger et al., 2024; Stein and Meredith, 1993). As a result, differences in sensory sensitivity, which occur in both ASD and ADHD, can masquerade as differences in multisensory integration. The present perception-matched approach does not assume that detection thresholds fully capture the broader construct of sensory processing; rather, it functions as a methodological control. By equating unisensory detectability across participants, this design reduces confounding influences of sensory sensitivity and allows audiovisual effects to be interpreted more specifically in terms of multisensory integration mechanisms.

### 2.4 Questionnaires

#### 2.4.1 ADHD adult self-report scale

To assess participants’ experiences with ADHD symptoms and frequency of symptoms, the ADHD Adult Self-Report Scale (ASRS; Kessler et al., 2005) was used. There is a total of 18 questions in the scale to determine the frequency of ADHD symptoms based on DSM-IV Criterion A symptoms (American Psychiatric Association, 2013). The scale included two subscales, each with nine questions: inattention and hyperactivity/impulsivity subscale. A five-point Likert scale is used in the ASRS, ranging from zero, “never,” to four, “very often” (Kessler et al., 2005). A high probability of an ADHD diagnosis is predicted by a score of 47 or higher. The scale’s reliability (Cronbach’s 𝛼 of 0.82) is satisfactory (Reuter et al., 2006).

#### 2.4.2 Broad autism phenotype questionnaire

To assess features of the Autism phenotype, the Broad Autism Phenotype Questionnaire was utilized (BAPQ; Hurley et al., 2007). Specifically, features of Autism associated with personality and language traits were assessed (Hurley et al., 2007). The BAPQ is an optimal assessment for Autistic traits in non-clinical or general population adults, given that it is specifically used to identify subclinical levels of definitive Autistic traits. The BAPQ was therefore chosen over other measures of Autistic traits, such as the Social Responsiveness Scale for example, which has been suggested to lack high specificity in adults and sometimes reflect general social difficulties that are not unique to Autism (Lampinen et al., 2025). The BAPQ assesses three elements of the broad Autism phenotype: *aloofness*, *rigid personality*, and *pragmatic language* deficits (Hurley et al., 2007). Aloofness is a form of social detachment that is recognized as shyness or disinterest. Rigid personality is defined as finding it challenging to adapt to change. Pragmatic language assesses challenges in verbal and non-verbal aspects of language that result in impaired communication in social settings. There are 36 self-report statement items in the questionnaire that are rated on a six-point Likert-type scale, ranging from “rarely applies” to “often applies” (Hurley et al., 2007). The scale has a high internal consistency as indicated by the Cronbach’s a coefficient of 0.94, 0.91, and 0.85 for aloof, rigid, and pragmatic language subscales, respectively (Hurley et al., 2007). As demonstrated by previous studies, the reliability of the BAPQ (>70%; Hurley et al., 2007) is based on sensitivity and specificity requirements (Sasson et al., 2013; Seidman et al., 2012).

### 2.5 Analysis

#### 2.5.1 Threshold validation

Threshold values were determined by the three staircases in each domain that converged by taking the median level of the three staircases after six reversals were made within each staircase in each sensory domain. To validate the threshold values to confirm they accurately reflect each participant’s 50% threshold value, we calculated the auditory and visual detection rates individually across trials during the second phase and conducted a single-sample *t*-test with an expected value of 0.5, which were calculated using SPSS (version 29). Participants with unisensory accuracy below 40% or above 60% were removed (*N* = 32).

#### 2.5.2 Response time analysis

For each participant, multisensory gain was calculated using response times in the speeded detection task using the Race Model and Miller’s Inequality (Miller, 1982; Raab, 1962). The MATLAB RSE-box (Otto, 2019) was used to calculate the race model, the response-time based multisensory gain. The race model uses cumulative distribution functions (CDF) to represent the cumulative probability that a response has been made at a given time (Miller, 1982; Raab, 1962). Miller’s bound was used as a baseline for identifying multisensory gain, accounting for statistical facilitation due to redundant target effects and assumes a maximum negative correlation between auditory and visual response times, making it a conservative baseline (Miller, 1982).

Race model violations are identified when the observed audiovisual CDF crosses to the left of Miller’s bound. Violations of Miller’s bound indicate that a multisensory interaction has occurred, and the difference between the observed audiovisual CDF and Miller’s bound is indicative of how much multisensory gain is experienced. Race Model violations occur when the actual RTs to audiovisual stimuli are faster than the predicted reaction times, which results in a positive value. Positive values are interpreted as evidence of multisensory integration. Conversely, the absence of violations, or values of zero, cannot be conclusively interpreted as evidence for or against multisensory integration. This lack may suggest sub-optimal integration, independent processing, or interference instead (Stevenson et al., 2014a). The RSE-box reports the Violation of Miller’s bond as a singular number, which we will report. Other methods of calculating race model provide different information, such as time bins.

#### 2.5.3 Accuracy

Accuracy for auditory, visual, and audiovisual trials will be reported. Further, accuracy gain will be measured using the probability summation criteria where predicted audiovisual accuracy will be subtracted from observed audiovisual trial accuracy. We used the probability summation criteria rather than comparing audiovisual performance to a maximum unisensory condition, because while the maximum unisensory approach tests whether audiovisual performance exceeds the better of the auditory or visual conditions, it does not account for statistical facilitation arising from redundant signals. When auditory and visual inputs are processed independently, the presence of two signals increases the likelihood that at least one will be detected, leading to improved performance even in the absence of true integration. Probability summation explicitly models this independence assumption by estimating the audiovisual response rate expected from parallel, independent processing of unisensory (e.g., auditory and visual) inputs. Thus, only audiovisual performance exceeding this predicted value reflects facilitation beyond statistical redundancy and provides evidence for multisensory integration (Stevenson et al., 2014a).

Predicted audiovisual accuracy was calculated from the unisensory component accuracy, assuming independence using the following equation:


pAV=p(A)+p(V)−[p(A)∗p(V)]

Where *pAV* represents the null hypothesis of the response to audiovisual presentations if the auditory and visual information are processed independently, and where *p(A)* and *p(V)* represent response accuracy to auditory- and visual-only presentations. Multisensory accuracy gain was then derived using the following equation: 𝑜𝑏𝑠𝑒𝑟𝑣𝑒𝑑 𝐴𝑉 𝑎𝑐𝑐𝑢𝑟𝑎𝑐𝑦− 𝑝Â𝑉^t^ 𝑎𝑐𝑐𝑢𝑟𝑎𝑐𝑦. Positive values indicated that multisensory integration had occurred, while negative values failed to conclude that multisensory integration had occurred.

#### 2.5.4 Correlations

Pearson’s *r* correlation analyses were used to examine the relationships between multisensory gain, unisensory accuracy, multisensory accuracy and Autistic and ADHD traits. If data was non-normally distributed, we used Spearman’s rho correlations.

#### 2.5.5 Moderation

A moderation analysis was conducted using Model 1 of the PROCESS macro (Hayes, 2022) to examine whether ADHD traits (total ASRS score) moderate the association between Autistic traits (BAPQ total score) and accuracy gain. A moderation model was conducted with Autistic traits (as measured through the BAPQ) as the predictor and ADHD traits (as measured by the ASRS) as the moderator to predict measures of multisensory gain (i.e., Miller’s race model violations and accuracy gain). ADHD traits were chosen as the moderator as a high rate of Autistic individuals have ADHD (Hours et al., 2022), but a lower rate of individuals with ADHD are Autistic (Hollingdale et al., 2020).

## 3 Results

### 3.1 ADHD and autistic traits

Total scores on the BAPQ ranged from 45 to 169 (*M* = 107.01, SD = 21.32). 23 participants (17 females) had an average total score above the identified self-reported cutoffs scores considered high for Autistic traits (3.55 standardized or 126 for males and 3.17 standardized or 114.12 for females; Sasson et al., 2013). Scores on the ASRS ranged from 4 to 57 (*M* = 32.33, SD = 9.70), with 8 participants (5 females) having a score of 47 or over, which is considered to indicate “most likely to have ADHD” (Stark et al., 2011).

### 3.2 Response time analysis

**Figure 2:**
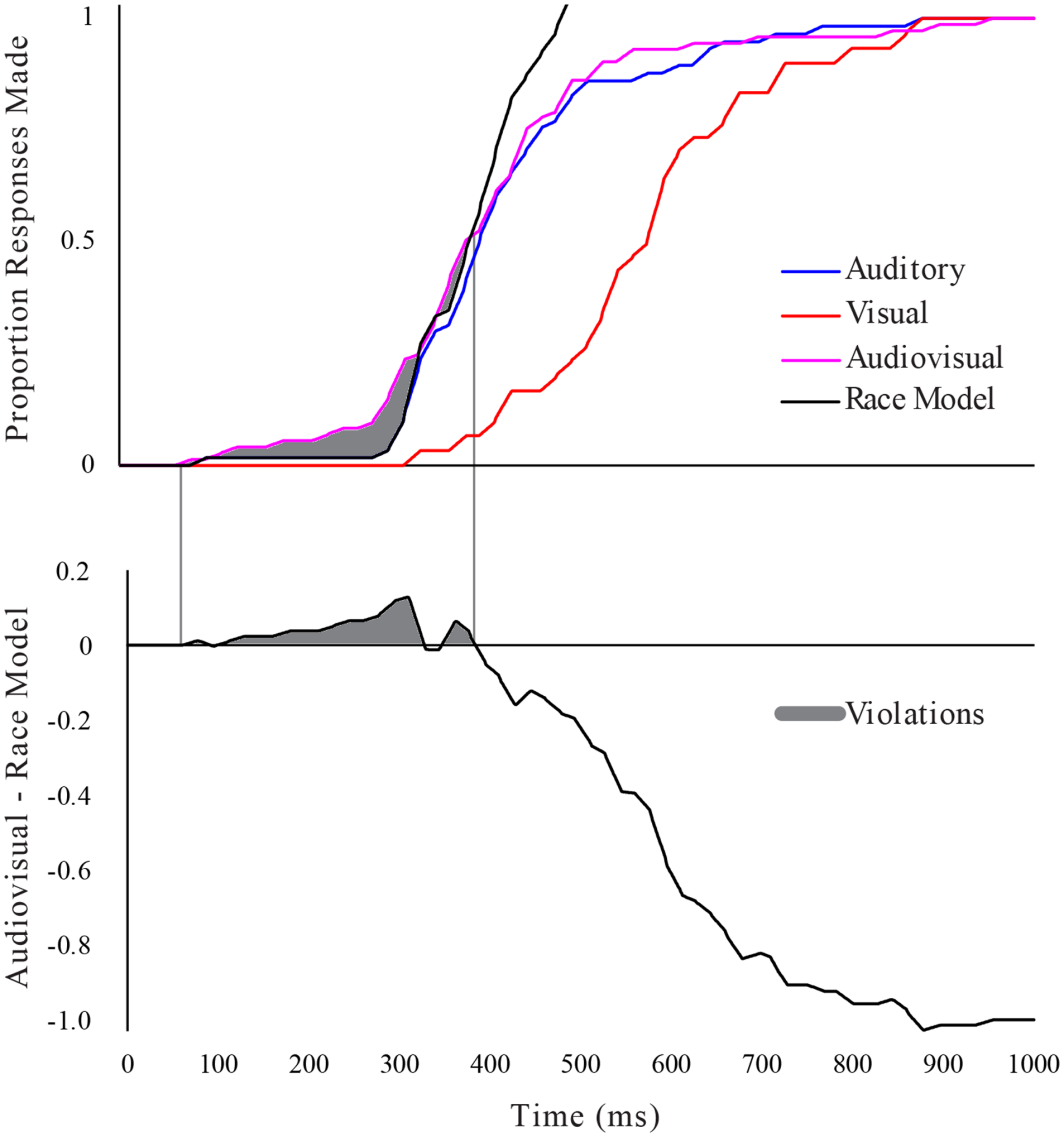
Example Violation of Race Model for an Example Participant. Note. Top panel includes cumulative distribution functions of response times in the three stimulus conditions (Auditory, Visual, Audiovisual) in comparison to Miller’s Race Model cumulative distribution. Bottom panel includes the violation represented by the shaded area. Note that only positive values constitute violations. Summed area under the curve is indexed as overall multisensory gain.

For response time gain, violations of Miller’s Race Model (*M* = 1.98, SD *=* 3.25) ranged from 0 to 14.62, with positive values suggesting a violation of Miller’s Race Model and evidence for multisensory integration (Figure 2).

### 3.3 Accuracy and accuracy gain

**Figure 3:**
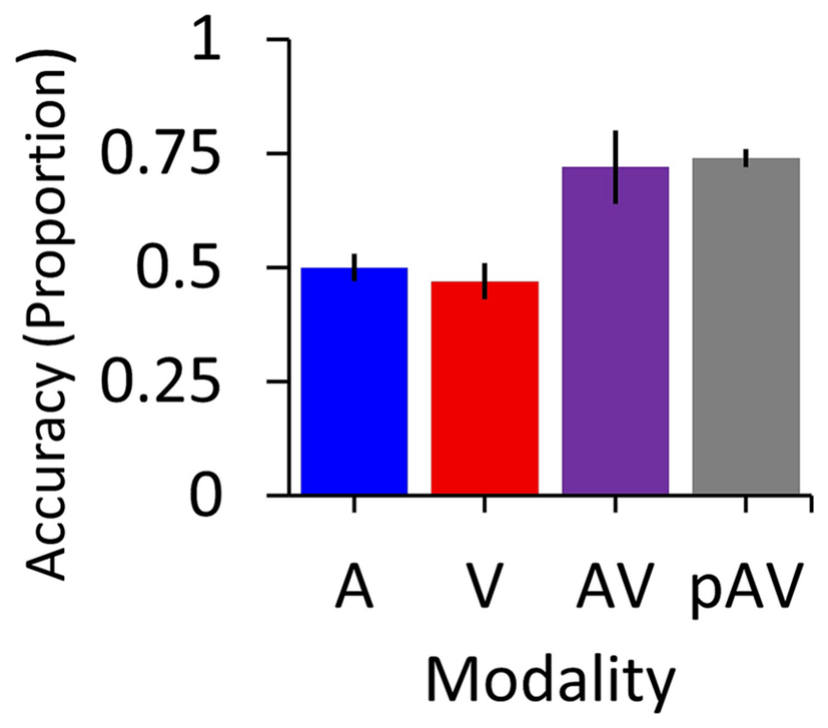
Mean accuracy for auditory, visual, and audiovisual stimuli and the probability summation criteria for all participants. Error bars indicate standard deviation.

Auditory, visual, audiovisual accuracy means, standard deviations and correlations with Autistic and ADHD traits are reported in Table 1. Accuracy gain (*M* = −0.02, SD = 0.08, range = −0.27, 0.13) was determined using the conservative probability summation criteria (Figure 3). Positive values indicated that multisensory integration had occurred, while negative values failed to conclude that multisensory integration had occurred. Overall, we did not find evidence of multisensory integration in the sample using the probability summation criteria.

**Table 1:** Means, SD, and correlations of accuracy and ADHD and autistic traits.

Trait subscale	Auditory accuracy			Visual accuracy			Audiovisual accuracy		
	*M*	*SD*	*CI*	*M*	*SD*	*CI*	*M*	*SD*	*CI*
	0.50	0.03		0.47	0.04		0.72	0.08	
	*r*	*p*		*r*	*p*		*r*	*p*	
ADHD									
Inattention	−0.02	0.87	[−22, 0.19]	0.13	0.23	[−0.08, 0.32]	0.15	0.16	[−0.06, 0.34]
Hyper-Imp	−0.21*	0.04	[−0.40, −0.01]	0.24*	0.03	[0.03, 0.42]	0.14	0.19	[−0.07, 0.33]
Total	−0.13	0.20	[−0.33, 0.07]	0.21*	<0.05	[0.002, 0.40]	0.16	0.12	[−0.05, 0.35]
Autistic									
Aloof	−0.07	0.53	[−0.27, 0.14]	0.06	0.11	[−0.14, 0.26]	0.15	0.16	[−0.06, 0.34]
Pragmatic Language	−0.22*	0.03	[−0.41, −0.02]	−0.01	0.94	[−0.21, 20]	0.12	0.24	[−0.04, 0.36]
Rigidity	−0.15	0.17	[−0.34, 0.06]	0.23	0.23	[−0.08, 0.32]	0.22*	0.03	[−0.17, 0.24]
Total	−0.08	0.47	[−0.28, 0.13]	−0.003	0.98	[−0.21, 20]	0.20	0.06	[−0.01, 0.39]

*N* = 92 *p < 0.05.

### 3.4 Correlations

Correlations for violations were run twice, first, correlations were run for all participants and second, correlations were run for only participants showing evidence of multisensory integration using Miller’s Race Model (Violations > 0) (Table 2). As the data was non-normally distributed for violations, we conducted a Spearman rho correlation between violations of Miller’s Race Model and ASRS and BAPQ scores and 1 outlier was removed. Autistic and ADHD traits were not significantly related to violations of Miller’s Race Model for all participants and those with evidence of multisensory integration.

**Table 2:** Correlations between autistic and ADHD traits and violations of millers race model with all participants and only violations showing evidence of multisensory integration (violation > 0).

Trait subscale	Total sample (*N* = 57)		Race model (*N* = 91)			Race model (*N* = 56)		
	*M*	*SD*	*ρ*	*p*	*CI*	*ρ*	*p*	*CI*
ADHD								
Inattention	18.09	5.93	−0.16	0.14	[−0.36, 0.06]	−0.14	0.29	[−0.40, 0.13]
Hyper-Imp	13.63	5.49	−0.02	0.87	[−0.23, 0.20]	−0.01	0.94	[−0.28, 0.26]
Total	31.72	10.36	−0.07	0.50	[−0.28, 0.14]	−10	0.45	[−0.36, 0.17]
Autistic								
Aloof	35.28	9.38	−0.007	0.95	[−0.22, 0.21]	0.21	0.12	[−0.07, 0.45]
Pragmatic Language	33/32	7.28	−0.02	0.82	[−0.24, 0.19]	0.08	0.58	[−0.20, 0.34]
Rigidity	37.74	8.32	0.01	0.92	[−0.20, 0.22]	0.09	0.52	[−0.19, 0.35]
Total	106.33	21.24	−0.02	0.88	[−0.23, 0.20]	0.14	0.29	[−0.13, 0.40]

**Figure 4:**
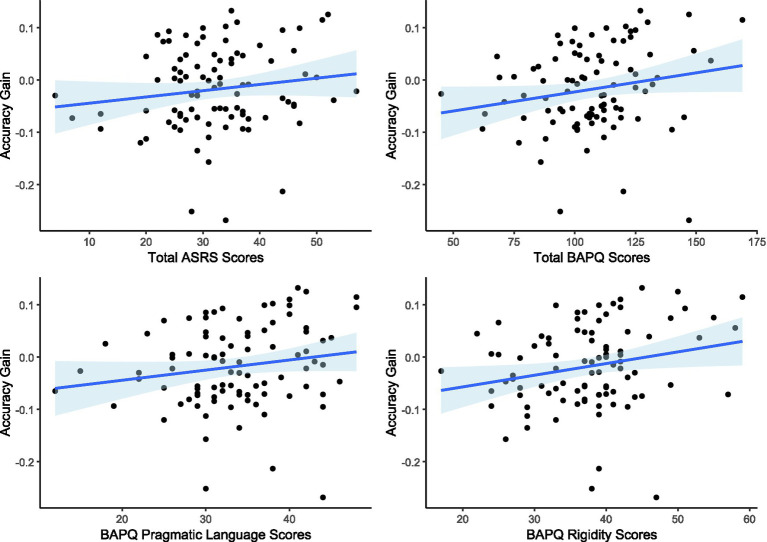
Correlations between accuracy again and total ASRS scores, total BAPQ, and pragmatic language and rigidity subscales (*N* = 92). ASRS, Adult ADHD Self-Report Scale; BAPQ, Broad Autism Phenotype Questionnaire.

Pearson r correlations between accuracy gain determined by the probability summation criteria, and ASRS and BAPQ scores are reported in Table 3 and Figure 4. BAPQ rigidity was significantly positively related to accuracy gain but did not survive multiple corrections. There was not a significant correlation between accuracy gain and ASRS scores.

**Table 3:** Correlations between autistic and ADHD traits and accuracy gain.

Trait subscale	Total sample (*N* = 92)		Accuracy gain (*N* = 92)		
	*M*	*SD*	*r*	*p*	*CI*
ADHD					
Inattention	17.96	5.55	0.13	0.23	[−0.08, 0.32]
Hyper-Imp	13.55	5.55	0.13	0.21	[−0.08, 0.33]
Total	31.51	9.53	0.15	0.17	[−0.02, 0.32]
Autistic					
Aloof	35.73	9.81	0.11	0.29	[−0.18, 0.18]
Pragmatic Language	33.77	7.57	0.17	0.10	[−0.12, 0.23]
Rigidity	37.70	8.38	0.23*	0.03	[−0.07, 28]
Total	107.19	21.79	0.20	0.06	[−0.12, 24]

*p < 0.05.

### 3.5 Moderations

#### 3.5.1 Response time

A moderation analysis using Model 1 of the PROCESS macro for SPSS (Hayes, 2022) was conducted to test whether ADHD traits (ASRS total score) moderated the relationship between Autistic traits (BAPQ total scores) and response time violations. The model included BAPQ total score as the predictor (X), ASRS total score as the moderator (W), and violations as the outcome variable (Y). The sample consisted of 56 participants with violations showing evidence of multisensory integration.

The overall model was not statistically significant, *F*(3, 52) = 2.30, *p* = 0.09, though did explain approximately 11.7% of the variance in violations (*R*^2^ = 0.12). The main effect of BAPQ total scores on violations was not significant [*b* = −0.02, SE = 0.07, *p* = 0.74, 95% CI (−0.1643, 0.1181)]. Similarly, the main effect of ASRS total scores on violations was not significant, though approached significance [*b* = −0.34, SE = 0.1912, *p* = 0.08, 95% CI (−0.7250, 0.0421)]. The interaction between BAPQ and ASRS scores was also not statistically significant [*b* = 0.0026, SE = 0.0020, *p* = 0.19, 95% CI (−0.0013, 0.0065)], indicating that ADHD traits did not moderate the relationship between Autistic traits and violations. The interaction term accounted for an additional 2.96% of variance in the outcome, which was not significant, *F*(1, 52) = 1.74, *p* = 0.19.

#### 3.5.2 Accuracy gain

The model included BAPQ total score as the predictor (X), ASRS total score as the moderator (W), and accuracy gain as the outcome variable (Y). The analysis was conducted with 92 participants.

The overall model was not statistically significant, *F*(3, 88) = 1.33, *p* = 0.27, accounting for approximately 4.34% of the variance in academic gain (*R*^2^ = 0.04). Neither the main effect of BAPQ total score [*b* = 0.0003, SE = 0.0012, *p* = 0.21, 95% CI (−0.0022, 0.0027)] nor the main effect of ASRS total score [*b* = − 0.0004, SE = 0.0034, *p* = 0.91, 95% CI (−0.0071, 0.0063)] were statistically significant predictors of accuracy gain. The interaction term between BAPQ total score and ASRS total score was also not statistically significant [*b* = 0.0000, SE = 0.0000, *p* = 0.76, 95% CI (−0.0001, 0.0000)], indicating no evidence that ADHD traits moderated the association between BAPQ traits and accuracy gain. The interaction explained an additional 0.1% of variance in the outcome, which was not significant, *F*(1, 88) = 0.93, *p* = 0.76.

## 4 Discussion

Multisensory integration differences have been reported in both Autistic and ADHD populations, yet these conditions are rarely examined together despite co-occurrence estimates suggesting substantial overlap. The present study therefore sought to disentangle the unique and combined contributions of Autistic and ADHD traits to multisensory integration within a broad university sample using a perception-matched speeded detection task. Contrary to our hypotheses, multisensory integration was not associated with either Autistic or ADHD traits, nor was there evidence of an additive or interactive effect when both trait levels were elevated. This pattern is somewhat inconsistent with prior work using speeded audiovisual detection paradigms, which has reported considerable variability in behavioural outcomes. In clinical ASD samples, behavioural indices of multisensory integration in speeded detection tasks have shown mixed results across studies, with some reporting reduced integration and others observing no reliable differences (Dwyer et al., 2022; Molholm et al., 2020; Ostrolenk et al., 2019). In contrast, a study examining Autistic traits in a non-clinical adult sample reported slower responses to audiovisual stimuli, although these effects were interpreted in terms of heightened sensory reactivity rather than altered multisensory integration, as stimuli were not perceptually matched (Poulain et al., 2023). In ADHD, earlier violations of Miller’s Race model have been found in adults with ADHD (McCracken et al., 2019), but these effects may not directly translate to trait-level variation found in non-clinical samples. As highlighted in classic work on redundant-target paradigms (Townsend and Ashby, 1983; Townsend and Nozawa, 1995), race-model violations are highly sensitive to baseline perceptual differences and require careful matching of unisensory conditions, which may vary systematically across clinical and trait-level populations. Together these results suggest that multisensory integration may not be related to Autistic and ADHD traits, as measured specifically by accuracy gain and race model violations in perception-matched paradigm speeded detection task with simple stimuli.

Looking to multisensory integration work using differential paradigms, these findings diverge from trait-based research indicating reduced multisensory gain among individuals with higher Autistic traits (van Laarhoven et al., 2019) and from several studies of clinically diagnosed Autistic samples reporting attenuated gain (Collignon et al., 2013; Feldman et al., 2018; Ostrolenk et al., 2019; Stevenson et al., 2014d). At the same time, our results align with a subset of studies that have observed no differences in multisensory gain between Autistic and non-Autistic individuals (Baum et al., 2015; Bebko et al., 2014; Beker et al., 2018; Foxe et al., 2015; Stevenson et al., 2014b, 2017). In the ADHD domain, our findings are consistent with evidence from non-clinical and some clinical samples suggesting no association between ADHD traits and multisensory integration (Hare et al., 2024; Hare et al., 2026b; Schulze et al., 2021), but contrast other work in clinical ADHD populations showing increases in multisensory gain, particularly for response time-based indices (Hare, 2024; McCracken et al., 2019).

A key consideration in interpreting these discrepancies is the distinction between dimensional traits and clinical diagnoses. Much of the literature reporting robust multisensory integration differences has focused on clinically defined ASD or ADHD groups, where symptoms are more severe, pervasive, and functionally impairing. Overall, the absence of independent or combined trait effects suggests that multisensory integration differences may not scale linearly with subclinical Autistic and ADHD traits. Multisensory integration differences may only emerge beyond a threshold of symptom severity, reflect qualitative changes associated with diagnosis, or vary across developmental stages, given that multisensory processes continue to mature across childhood and adolescence. Accordingly, the absence of associations in the present adult, predominantly non-clinical sample should not be taken as evidence against multisensory differences in clinical or younger populations.

Methodological factors possibly further constrained the detectability of trait effects. The perception-matched detection paradigm was designed to control for individual differences in unisensory sensitivity, strengthening interpretability by minimizing confounds related to inverse effectiveness or modality dominance. However, this approach may also reduce between-participant variability that could otherwise relate to trait measures, and it remains unclear whether it is sufficiently sensitive to detect known group differences. In addition, the use of Gabor patches and beeps likely constrained the engagement of higher-order perceptual, temporal, and attentional mechanisms that are more reliably implicated in ASD and ADHD in studies using complex or speech-based stimuli. Future work should therefore validate perception-matched detection paradigms in clinically diagnosed and developmentally younger samples and examine whether trait-related effects emerge under conditions involving greater stimulus complexity or ecological validity. Overall, our null findings may reflect that there is no relationship between multisensory integration and Autistic and ADHD traits in the current study, or alternative interpretations of these results remain possible given the specific characteristics of the paradigm (e.g., perception-matched design, simple stimuli, and sample composition).

The results from the present study do not align with our prediction that multisensory integration will be negatively related to higher Autistic traits. We had predicted that individuals with higher Autistic traits would demonstrate lower multisensory gain compared to those with lower Autistic traits. This would have been consistent with studies that have investigated differences in multisensory integration between Autistic and non-Autistic individuals, which have found evidence for reduced integration in Autistic compared to non-Autistic individuals (Collignon et al., 2013; Feldman et al., 2018; Noel et al., 2017; Stevenson et al., 2014d). It is possible that previous studies using stimulus-matched stimuli were influenced by differences in sensory sensitivity across groups, and that when accounting for these differences, we may have minimized the expected and previously found relationship between Autistic traits and multisensory integration. We also found that several of our participants did not show evidence of multisensory integration using Miller’s race model violations or the conservative probability summation criteria for accuracy gain. This pattern is not uncommon, even in neurotypical samples, and may reflect both the stringency of the metrics used and the nature of the stimuli. Further, our study used simple stimuli (e.g., Gabor patches and beeps), for Autistic individuals differences are sometimes enhanced when using higher-level speech stimuli (Baum et al., 2015; Bebko et al., 2014; Beker et al., 2018; Foxe et al., 2015; Stevenson et al., 2014d, 2017) compared to lower level stimuli. The combination of simple stimuli and conservative criteria, both of which require relatively efficient integration to demonstrate effects, may therefore have contributed to the reduced evidence of multisensory integration observed at the group level, effectively diluting overall effects. With that said, restricting our correlational analysis to only participants who demonstrated race-model violations did not improve our effect sizes.

There were no significant correlations between the aloof and pragmatic language subscales of the BAPQ and either measure of multisensory gain. This could be due to the nature of what these subscales measure. The aloof subscale is meant to assess aloof personality, which is defined as a lack of interest in or enjoyment of social interactions (Hurley et al., 2007). While this measure is consistent with assessing Autistic traits, we did not assess interest in or enjoyment of social interactions in this task. The pragmatic language subscale assesses deficits in social aspects of language that may result in difficulties in social communication (such as difficulties with effectively holding a fluid, reciprocal conversation; Hurley et al., 2007). Thus, it is possible that no significant correlations were found between our measures of multisensory gain and this subscale, as our task assessed very basic level measures of sensory perception, which may not be impacted by higher order cognitive processes, such as social interactions. Previous research that has found significant associations with the BAPQ and multisensory integration has in fact used stimuli assessing higher order cognitive processes to assess these relationships, such as the McGurk task which assesses multisensory integration in speech processing (Feldman et al., 2022).

Interestingly, we did find a significant correlation between the rigidity subscale of the BAPQ and multisensory gain; however, only when assessing accuracy, not reaction times. However, these results should be interpreted with caution as they did not survive multiple comparisons. This result was likely driven by the observed higher accuracy for audiovisual trials, and not lower accuracy for the unisensory stimuli. The rigidity subscale assesses rigid personality, which is defined as little interest in change or difficulty adjusting to change (Hurley et al., 2007). This subscale is said to address the stereotyped and repetitive behaviours seen in Autistic individuals. While differences in sensory perception are classified under this category in the diagnostic criteria for ASD, limited items of this subscale actually assess sensory processing. Given the characteristic of rigidity, it may be thought to be related to a reduced tolerance for sensory ambiguity or difficulty updating sensory predictions. Perhaps since our sample is predominately non-clinical with only 23 participants met the cut-off for high Autistic traits according to the BAPQ, these participants may have been very diligent in doing the task.

As previously mentioned, the relationship between ADHD and multisensory integration has been inconsistent. We hypothesized that response time-based measure of multisensory gain would be positively related to ADHD traits. Previous research showed enhanced multisensory integration using Miller’s Race Model, with one showing an earlier violation in adults with ADHD compared to NT adults (McCracken et al., 2019), and another showing a larger magnitude of violations in adults with ADHD (Hare, 2024). However, our study found no significant relationship between multisensory gain and ADHD traits, though it should be noted that even with a reduced sample size due to removal of individuals showing no violations this relationship did account for a marginally-significant 11.7% of variance. There are several potential explanations for this. First, it may be necessary to reach a clinical threshold to observe differences in multisensory integration in ADHD. In our sample, only eight participants scored above the suggestive diagnostic cut-off for ADHD, which may have limited our ability to detect meaningful relationships. Supporting this, another study investigating ADHD traits and multisensory integration in university students across three common tasks (i.e., sound-induced flash illusion, McGurk effect, and speech-in-noise) similarly found no significant relationship (Hare et al., 2026b), while these tasks have shown differences in clinical ADHD populations (Hare et al., 2024; Michalek et al., 2014; Schulze et al., 2021). Second, stimulus complexity might play a critical role. Our study used basic stimuli (flashes and beeps), while more pronounced differences in ADHD populations have been observed with complex stimuli, particularly speech-related tasks (Hare et al., 2024; Schulze et al., 2021). Third, our task was perception-matched and controlled for differences in sensory sensitivity. This approach may have minimized the expected differences in multisensory integration between individuals with varying levels of ADHD traits. No differences for subtype traits and multisensory integration were present. Previous research has found a stronger relationship between hyperactive–impulsive traits and multisensory processing compared to inattentive traits for a McGurk task and for temporal binding windows (Hare et al., 2024; Hare et al., 2026a). Hyperactive–impulsive traits were negatively related to auditory accuracy but positively related to visual accuracy. ADHD or more broadly, attentional problems or distractibility, seem to be associated with auditory filtering and processing challenges (Cheung and Siu, 2009; Conzelmann et al., 2009; Ghanizadeh, 2011; Hutchison et al., 2017).

We hypothesized that high Autistic and ADHD traits together would be related to reduced multisensory integration, but we did not find an interaction between Autistic and ADHD traits. The combined effects of Autism and ADHD on multisensory integration may not follow a simple additive pattern, and this could explain why the two conditions together do not necessarily exacerbate difficulties in multisensory integration for nonclinical populations. Although ADHD and ASD are often comorbid, their effects on multisensory processing appear to follow distinct patterns, which may offset or neutralize one another when co-occurring. Interestingly when looking at sensory processing more broadly, sensory phenotypes characterizing sensory processing showed similar patterns of association with features of Autism and ADHD, across both diagnostic groups (Scheerer et al., 2024). Focusing on clinical samples where traits are more pronounced may provide clearer insights into these interactions. Future studies examining multisensory integration in populations with both ASD and ADHD may benefit from having an Autistic group, ADHD group, and combined group to examine whether the comorbid group is more similar to the ADHD or Autistic group.

### 4.1 Limitations and future research directions

There are a number of limitations to consider when interpreting our results. First, we did have a greater number of participants with high Autistic traits (*n* = 23) compared to high ADHD (*n* = 8) traits. It is possible that this may have impacted our results and contributed to the significant correlation on the one BAPQ subscale and not on the ASRS subscales. Future research may include a more representative sample of individuals with high Autistic and ADHD traits to determine whether this impacts the relationships between these traits and the multisensory gain. There is also a lack of evidence that multisensory integration occurred using the probability summation criteria for accuracy gain. Future research should validate this paradigm in clinical populations. Another limitation is the task assessed lower order cognitive processes; however, the measures we used included subscales that assessed higher order cognitive processes, which may be why we found no significant correlations. Future research should investigate the relationship between tasks using more complex stimuli, such as speech. Finally, it is possible that we found no significant moderation due to the fact that our sample was primarily university students. In typical development, multisensory integration is slower to develop than many unisensory processes (Ernst, 2008; Gori et al., 2008; Murray et al., 2016; Stevenson et al., 2018) and reaches maturity later in development with estimates ranging from fourteen to adulthood (Brandwein et al., 2011; Stevenson et al., 2018). There is some evidence that suggests that atypical multisensory integration is more evident in adolescence, and some of these differences normalize by adulthood in Autism (Beker et al., 2018; Foxe et al., 2015). It is possible that in order to see an effect of traits on integration you need to reach clinical criteria or be earlier in multisensory integration development. Future research should look at multisensory integration in comorbid ASD and ADHD youth. Finally, the BAPQ was used in this study to assess Autistic traits, as it is an optimal assessment of Autistic traits in non-clinical adults. However, the BAPQ contains very few items that assess sensory differences specifically and therefore may have limited the relationships found in the present study.

Given that multisensory work is still in its infancy for both ADHD and the ADHD and ASD comorbidity, there are many avenues for future research beyond work in clinical populations, using more complex stimuli, and examining this developmentally. First, future research may also include a sensory specific questionnaire, such as the Adult Adolescent Sensory Profile (Brown and Dunn, 2002), to examine sensory specific trait differences in non-clinical adults, in addition to broader Autistic traits. Second, the adaptive paradigm could be adjusted to see what makes it the most sensitive and reliable, for example you could include more trails or adjust the stimulus threshold to 70% instead of 50%. Third, there is limited work looking at the neural correlations of multisensory integration in these populations. One interesting possibility is if there are no behavioural differences but yet neural differences exist, which may suggest that individuals have developed compensatory mechanisms to maintain more optimal multisensory integration. Last, a lot more work needs to be done to disentangle how ASD and ADHD differentially relate to multisensory integration, attentional allocation, and response variability, rather than assessing a single shared mechanism.

## Data Availability

The raw data supporting the conclusions of this article will be made available by the authors, without undue reservation.
